# Spraying dynamics in continuous wave laser printing of conductive inks

**DOI:** 10.1038/s41598-018-26304-9

**Published:** 2018-05-22

**Authors:** Pol Sopeña, Sergio González-Torres, Juan Marcos Fernández-Pradas, Pere Serra

**Affiliations:** 1 Department of Applied Physics, Universitat de Barcelona, Martí i Franquès 1, 08028 Barcelona, Spain; 2 Institute of Nanoscience and Nanotechnology (IN2UB), Universitat de Barcelona, Joan XXIII S/N, 08028 Barcelona, Spain

## Abstract

Laser-induced forward transfer (LIFT), though usually associated with pulsed lasers, has been recently shown to be feasible for printing liquid inks with continuous wave (CW) lasers. This is remarkable not only because of the advantages that the new approach presents in terms of cost, but also because of the surprising transfer dynamics associated with it. In this work we carry out a study of CW-LIFT aimed at understanding the new transfer dynamics and its correlation with the printing outcomes. The CW-LIFT of lines of Ag ink at different laser powers and scan speeds revealed a range of conditions that allowed printing conductive lines with good electrical properties. A fast-imaging study showed that liquid ejection corresponds to a spraying behavior completely different from the jetting characteristic of pulsed LIFT. We attribute the spray to pool-boiling in the donor film, in which bursting bubbles are responsible for liquid ejection in the form of projected droplets. The droplet motion is then modeled as the free fall of rigid spheres in a viscous medium, in good agreement with experimental observations. Finally, thermo-capillary flow in the donor film allows understanding the evolution of the morphology of the printed lines with laser power and scan speed.

## Introduction

During the last decades, there has been an increasing demand from diverse industrial sectors towards the manufacture of cost-effective electronic circuits on platforms different from the traditional silicon wafers^1,2^. The relatively new field of printed electronics uses well-established printing techniques borrowed from the graphics industry to deposit a plethora of materials following the regular patterns that ultimately constitute the layout of the devices required in the applications^3,4^. The versatility of these techniques allows the printing of circuits on both rigid and flexible substrates, either inorganic or organic, covering a broad range of materials that spans from glass or polymers to paper or fabric^5–8^.

The most widespread printed electronics techniques, such as roto-gravure, flexography or screen printing, rely on the use of rolls and screens in order to carry out printing^9–11^. This makes these techniques adequate for long runs, but they fall short when customized production or short runs are required, as well as in defect repair applications, since roll and screen production is expensive and time consuming. In this case, digital-printing techniques such as inkjet-printing^12,13^ constitute an attractive alternative. In fact, the interest of digital manufacturing is not limited to small-scale production. Thanks to its flexibility, it is already considered as the paradigm towards which even large-scale manufacturing should tend. Through inkjet-printing, many kinds of inks containing materials as diverse as metallic nanoparticles, dielectrics or dyes can be successfully deposited^14–16^. However, there are limitations concerning the rheological properties of the ink that can limit the scope of the technique in some instances. On one hand, the range of printable viscosities for a given printing head is quite narrow, typically between 1 and 50 mPa·s for most commercial units. On the other hand, there are also limitations affecting the size of the particles suspended in the ink: it is usually accepted that 1/100th of the output nozzle diameter (normally a few tenths of microns) constitutes the upper limit of the printable particle size^17^.

**Figure 1:**
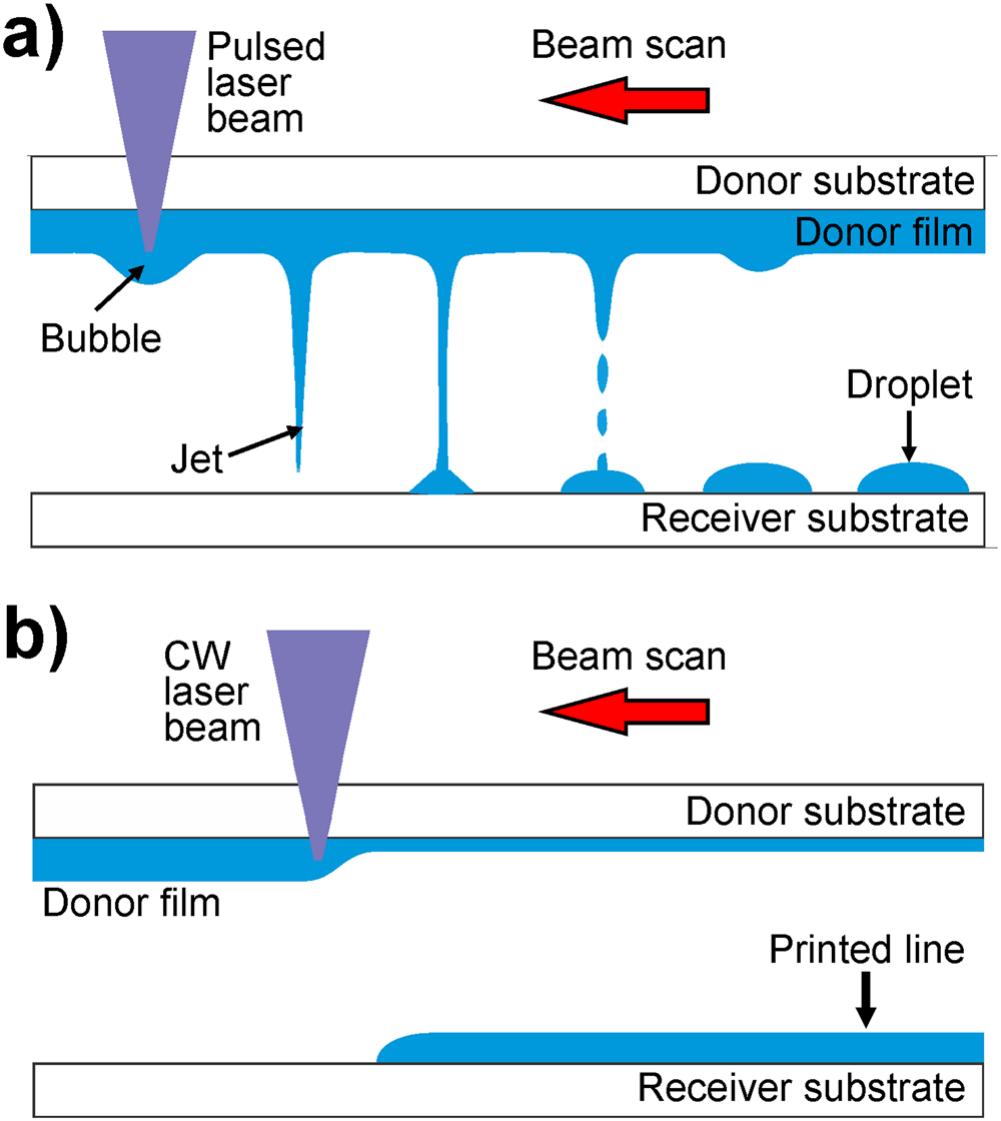
(**a**) Sketch of the mode of operation of the pulsed LIFT of liquids. The donor substrate covered with ink is displayed on the top and as the laser scans the sample at a certain repetition rate droplets are deposited on the receiver through a jetting dynamics. The different stages of transfer are represented: laser absorption, bubble expansion, jet formation and droplet deposition. (**b**) Sketch of the mode of operation of the CW-LIFT of liquids. The laser scan results in the deposit of a continuous line of ink on the receiver substrate.

Through the action of a laser pulse focused on a liquid film, usually called donor film, the laser-induced forward transfer (LIFT) technique allows transferring liquids from that film to a receiver substrate (Fig. 1a)^18–21^. The laser pulse is absorbed in the donor film, which leads to the generation of a bubble at the interface between the transparent donor substrate and the ink. The further expansion of the bubble results in the formation of a jet that propagates forward until it impacts the receiver substrate, and thus prompts the formation of a sessile droplet^22–26^. This process can be repeated multiple times at different locations on the donor film in order to generate the desired pattern^27–33^. Being nozzle-free, LIFT does not present the limitations of inkjet printing described above: a much broader range of loading particle sizes (at least up to 10 µm) and viscosities (from a few mPa·s to hundreds of Pa·s) are accessible with the technique^34–38^.

Traditionally, very short laser pulses, between nanoseconds and femtoseconds, have been used for the LIFT of liquids. However, in a previous study we already proved that material transfer with substantially longer pulses is possible^39^. This is clearly beneficial in economic terms: it is possible to achieve cost reductions of up to a 50% through the use of laser sources delivering pulses of several hundreds of nanoseconds, for example. Stretching the time scale to the ultimate level, we recently proved that it is possible to successfully print liquids by means of LIFT even with CW laser radiation^40^. In that work, we provided a proof-of-concept for the new approach through the fabrication of a functional sensing device entirely printed with CW-LIFT. Thus, the use of CW lasers results in an even higher economic impact than long-pulsed LIFT, especially taking into account that relatively low powers (on the order of 1 W) are enough for printing applications^40^. In addition, CW-LIFT allows using more compact and low maintenance lasers, with lower energy consumption and fewer safety issues.

In this paper, and building upon our previous work^40^, we extend the study of CW-LIFT to a substantially broader range of experimental conditions, focusing the attention on the understanding of the transfer dynamics and its underlying mechanisms, as well as on the correlation between that transfer dynamics and the printing outcomes. For that aim, we print silver nanoparticle (Ag-NP) ink lines on a glass substrate at different laser powers and scan speeds. Then, using a fast-photography setup we record movies of liquid ejection during CW-LIFT at the same conditions as those of the printing experiment. The characterization of the printed lines, alongside the analysis of the transfer dynamics revealed by the movies, allows us to approach the aimed understanding.

## Results and Discussion

Two main studies were carried out. The first one consisted on the analysis of the influence of the main process parameters on the morphology of the printed features, which was performed through the CW-LIFT printing of straight lines of Ag-NP ink at different laser powers and scan speeds and their further characterization. In the second one we analyzed the dynamics of liquid ejection during CW-LIFT through a fast photography imaging study of the process at the same printing conditions used in the former experiment. This allowed unveiling transfer dynamics completely different from that of traditional pulsed LIFT, and correlating it with the printing results.

### Line printing

**Figure 2:**
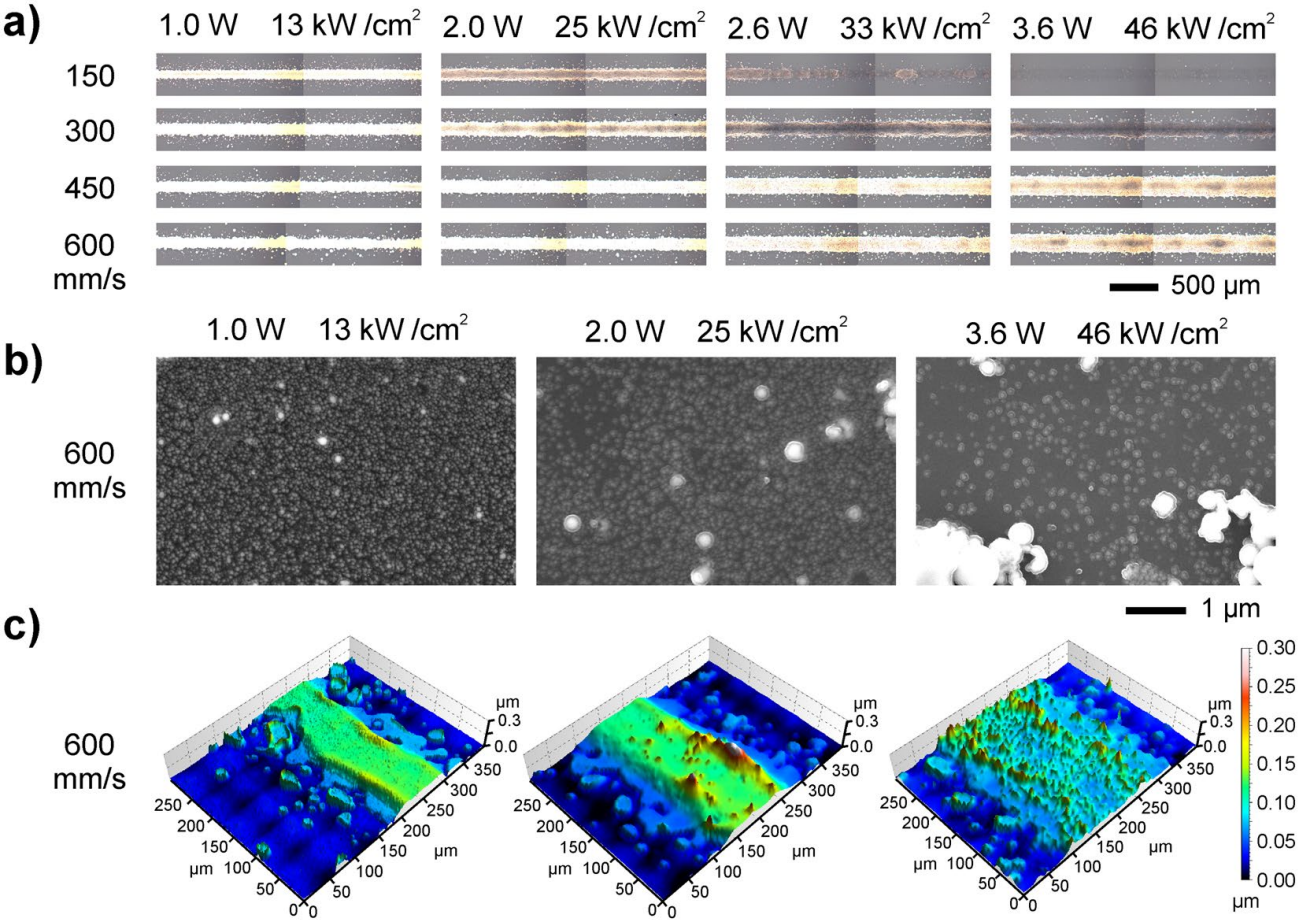
(**a**) Optical microscopy images of dry and sintered Ag-NP lines transferred at different laser powers (top) and scan speeds (left); the irradiation intensity is also provided (top). The total length of the printed lines is 2.5 cm. (**b**) SEM images of a portion of the center of the lines printed at 600 mm/s and different laser powers. The amount and size of NPs aggregates increases with laser power, whereas the concentration of single NPs decreases. (**c**) Confocal images corresponding to the selection presented in (**b**). Lines are wider and thinner as the laser power increases.

Straight lines of Ag-NP ink were printed following the procedure sketched in Fig. 1b at laser powers ranging between 0.5 and 3.6 W and scan speeds from 150 to 600 mm/s; optical microscopy images of the obtained results are presented in Fig. 2a. In most of the analyzed conditions, continuous lines as long as 2.5 cm are observed, with no bulging in any case. At 0.5 W (the minimum laser power, not shown in Fig. 2a), however, no continuous lines were obtained at any scan speed; only very small droplets arranged along the laser scan direction could be appreciated, similar to those observed at 3.6 W and 150 mm/s. The absence of bulging is relevant, since it is a major source of undesired short circuits between adjacent lines in printed devices. This phenomenon is a common problem in direct writing techniques like inkjet printing. It appears as a result of the printing mechanism, which consists on the forming of the lines through the overlap of successive droplets, and can be very detrimental in printed electronics applications^41,42^. CW-LIFT is free from bulging since printing occurs through the continuous ejection of material instead of through accumulation of sequentially transferred droplets. Nevertheless, the definition of the printed lines is not perfect; some satellite droplets (around 20 µm in diameter) appear alongside the edges. However, as proved in our previous study^40^ through the fabrication of a sensor containing a set of interdigitated electrodes, the satellites are not necessarily detrimental for the functionality of the resulting device: irrespective of the substrate used in that case (glass, polyimide, paper) no short circuit between adjacent electrodes was ever observed. Many commercial printed devices (sensors, RFID tags, etc.) have dimensions that do not require high levels of line definition^1,2,6,7^ and are not essentially different from the proof-of-concept provided in ref.^40^.

Three different types of morphology can be recognized depending on the printing conditions: bright and uniform lines, lines with a darkened center, and almost invisible traces. This last morphology (as in Fig. 2a at 3.6 W and 150 mm/s) corresponds to the practical absence of transferred material, though some scattered droplets are found in the receiving substrate under close inspection. At a scan speed of 150 mm/s in Fig. 2a, the three morphologies are present, with a clear evolution from bright/uniform to invisible traces as the laser power increases. The same evolution seems apparent at the other scan speeds, with the transition from one morphology to another occurring at higher powers as the scan speed increases. The line width ranges between 120 μm and 240 μm along the entire set of analyzed conditions; such feature sizes are similar to those typical of screen printed devices (RFID tags, sensors)^11,43^. Nevertheless, we can expect that the spatial resolution in CW-LIFT could be increased by tighter focusing of the laser beam on the donor film.

SEM images of the central region of each line were obtained, and a representative selection is shown in Fig. 2b. At low laser powers (1.0 and 2.0 W), Ag-NPs homogeneously cover the whole line and only some small aggregates of few NPs are observed. As the laser power increases, the aggregates tend to become larger and more abundant. This phenomenon has been previously observed in laser sintering experiments^44,45^, and attributed to the melting and coalescence of Ag-NPs during irradiation. Also, the NPs concentration in the line decreases with laser power, leading to completely scattered NPs and even some voids at the highest powers (2.6 and 3.6 W).

**Figure 3:**
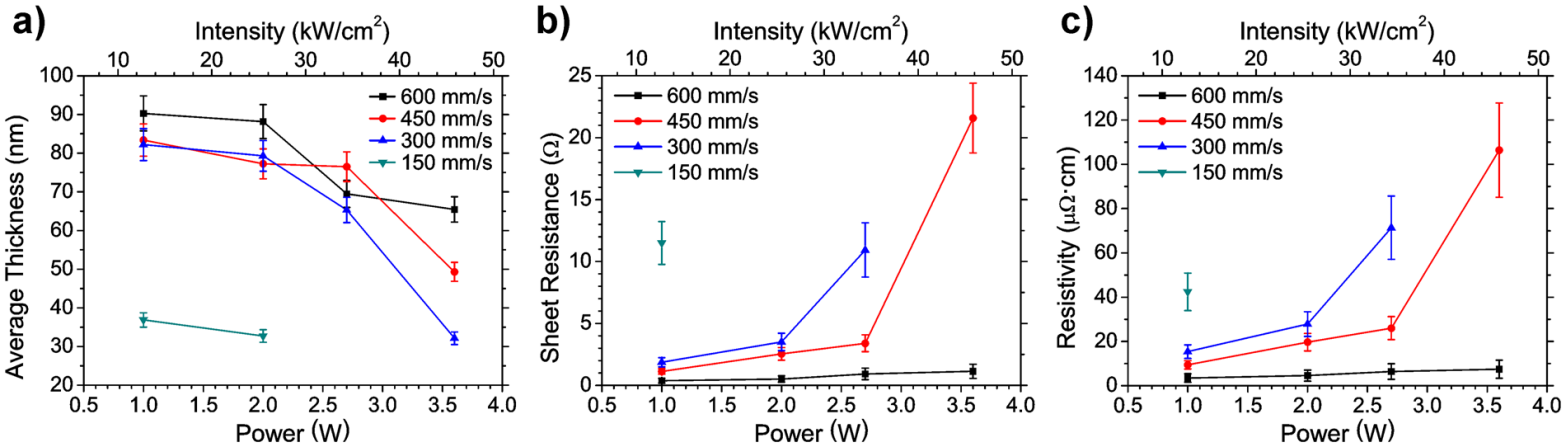
(**a**) Plot of the line thickness versus laser power at scan speeds of 150 (▼), 300 (▲), 450 (●) and 600 mm/s (■). Thickness decreases with increasing laser power for a given scan speed and, in general, increases with scan speed for the same power. (**b**) Plot of the sheet resistance of the lines versus the laser power at the same scan speeds as in (**a**). The sheet resistance increases with laser power for a given scan speed and decreases with scan speed for a certain power. (**c**) Plot of the resistivity of the printed ink versus the laser power at the same conditions as in (**a**) and (**b**). The resistivity trends with laser power and scan speed are similar to those of the sheet resistance.

Using confocal microscopy, 3D profiles of the lines were obtained, and a selection corresponding to the same conditions as those presented in the former paragraph is presented in Fig. 2c. At low laser powers (1.0 and 2.0 W) the transverse line profile is rather uniform, with a nearly rectangular cross section, but as the power increases material tends to accumulate in the line edges. At the highest powers (2.6 and 3.6 W), the center of the lines appears almost completely depleted (especially at speeds of 150 and 300 mm/s). The average line thicknesses were obtained from the 3D profiles, calculated as the ratio between the volume and the surface area provided by the microscope software (SensoMap 5.1). The corresponding results are plotted in Fig. 3a versus laser power for all the analyzed scan speeds. It is observed that line thickness tends to decrease with laser power. A maximum average thickness of 90 nm is obtained at 600 mm/s and 1.0 W, a value similar to that of lines deposited through inkjet-printing^14^. Also, in general terms line thickness seems to increase with scan speed at a fixed laser power. These data, together with the SEM images, can be related with the line uniformity and brightness distribution observed in the optical microscopy images. For instance, in the case of the laser scan speed of 600 mm/s, at low powers (1.0 and 2.0 W) the relatively uniform profile of the lines (Fig. 2c), as well as the rather homogeneous distribution of NPs (Fig. 2b), are consistent with the bright appearance of the lines in Fig. 2a. At the highest power (3.6 W), however, the lower concentration of Ag-NPs (plus scattered groups of aggregates, Fig. 2c) results in a less compact line, especially in the center, which accounts for its darker appearance. Similar agreements are found for the other laser scan speeds (Fig. 2a).

Electrical resistance measurements were carried out after curing in order to determine the optimum printing conditions for the aimed electronic applications. Sheet resistance versus laser power is plotted in Fig. 3b for all the analyzed scan speeds. It is observed that sheet resistance increases with laser power and decreases with scan speed. Remarkably, low sheet resistances, on the order of 1 Ω/□, are obtained at 600 mm/s, similar to those characteristic of inkjet printing^14^. At the scan speeds of 450 and 300 mm/s, sheet resistance increases dramatically above the laser power corresponding to the transition between bright/uniform lines and lines with dark center. At the lowest scan speed (150 mm/s) the printed lines are practically non-conductive. The corresponding resistivity was estimated from sheet resistance and average thickness measurements (assuming a uniform rectangular cross-section for all the lines). A minimum value of 3.5 μΩ·cm was obtained, 25% larger than the nominal value given by the provider, and approximately twice the value of bulk silver. The evolution of resistivity with laser power is similar to that of sheet resistance (Fig. 3c), but its relative increase at the laser power corresponding to the transition mentioned above is lower. In consequence, the increase observed in sheet resistance is not only due to the decrease in line thickness with laser power, a merely geometric effect, but it is also contributed by the increase in resistivity. This, in turn, can be attributed to the NP concentration in the line, which decreases with laser power, especially in the central part (Fig. 2b), the one that starts appearing darker at the transition. The influence of NP concentration on the obtained electrical resistivity can be due to the poor electrical contact among NPs at decreased concentrations, as well as to an overestimation of the filled volume fraction in the presence of voids, which translates into a higher apparent resistivity. A similar behavior has been reported for printed Ag lines showing a ‘coffee-ring effect’^46^.

From all these results it can be concluded that the optimal parameters for printing circuits correspond to lines deposited at high scan speeds and low laser powers, making 600 mm/s and 1.0 W the best scenario among the analyzed conditions, which is in good agreement with our previous study^40^. In these conditions, rather uniform and continuous lines with low sheet resistance are obtained. These results are especially attractive from an industrial point of view, since not only do they prove the feasibility of CW lasers, but also allow operating with low powers and high speeds. At higher speeds, and with tighter focusing of the laser beam, even better results could be expected. Although CW-LIFT had already been proved feasible from solid donor films^47^, the extension to liquids is remarkable, since this broadens considerably the range of materials printable with the technique; among others, conductive inks like the ones analyzed in this work, ubiquitous in printed electronics circuits.

### Transfer dynamics

**Figure 4:**
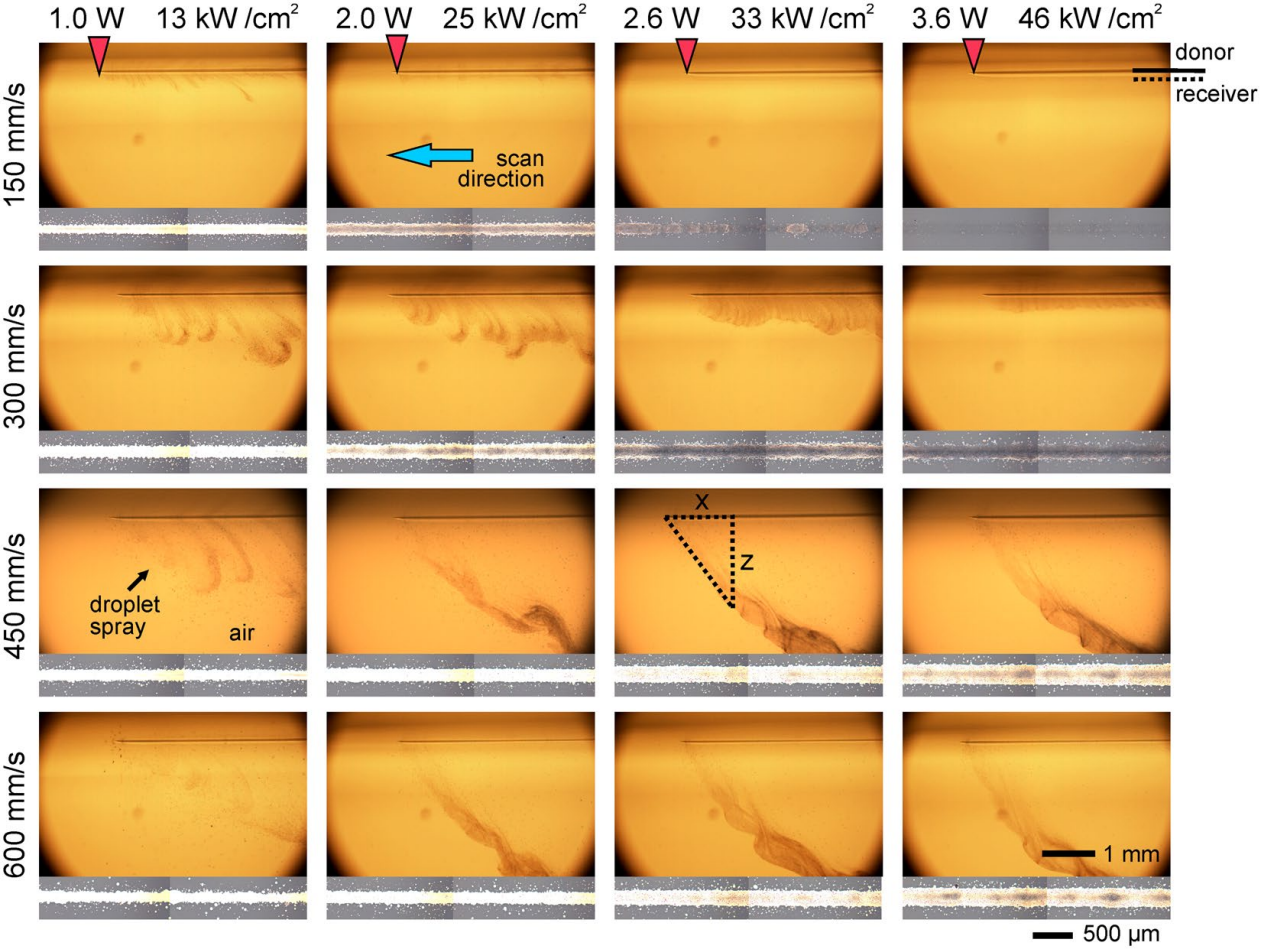
Selected frames of the transfer dynamics movies at different laser powers (top) and scan speeds (left); the corresponding printed line is shown below each frame. The donor film is located on the top of the frame, no receiver is present, and the laser scans the sample from right to left (blue arrow). On the top images a red arrow indicates the approximate position of the laser beam. The scale bar of 1 mm corresponds to the transfer dynamics movies and the scale bar of 500 μm corresponds to the printed lines. The position of the donor surface and receiver substrate during printing is indicated on the top-right end of the figure. The *x-z* diagram schematically represents the triangle method used to estimate the ejection speeds of Table 1. The full videos can be found in the Supporting Information.

Movies of the transfer process at different laser scan speeds and output powers were acquired using a fast-imaging camera, and a representative snapshot of an ejection event corresponding to each analyzed condition is presented in Fig. 4 (see Supporting Information for viewing the full videos). The donor film is always located on the top of the images, and the laser beam is scanned from right to left. The images were acquired without the receiver substrate to clearly visualize the ejection behavior.

It can be observed how, as the laser beam scans the donor film, ink is ejected as a myriad of tiny droplets that propagate in air like a spray. Three different types of ejection modes can be identified. The first one corresponds to a curtain of atomized ink in air (found at the lowest laser power for any scan speed, and at all laser powers for 300 mm/s; it is also barely visible at 150 mm/s and 2.0 W). The second one resembles a horsetail (at scan speeds of 450 and 600 mm/s for laser powers from 2.0 W onwards). Finally, the third one corresponds to the absence of visible ejection of material (scan speed of 150 mm/s at 2.6 and 3.6 W laser power). Remarkably, the observed behaviors are completely different from those of pulsed LIFT, in which transfer was achieved through the deposit of single droplets by a jet of ink. Instead, in CW-LIFT ink is transferred in a continuous manner as the laser beam scans the donor film.

The observed spraying dynamics can be attributed to pool-boiling in the donor film when it is irradiated by the laser beam^40^. In contrast to the typical picture of conventional pulsed LIFT, where a single cavitation bubble is generated at the donor substrate-film interface, in pool-boiling a myriad of bubbles would be produced in the irradiated area of the donor film, in the same way as in water boiling in a kettle. The longer irradiation times in CW-LIFT compared to those of pulsed LIFT are consistent with this hypothesis. Indeed, the time *t*_*i*_ that any portion of the donor film is irradiated by the laser beam can be estimated as:

1 $${t}_{i}=\frac{D}{v}$$

where *D* is the diameter of the laser beam on the sample and *v* the laser scan speed. For the scan speeds analyzed in this work (150–600 mm/s) these times range between 700 and 170 µs, much longer than the few ns irradiation times characteristic of pulsed LIFT. Furthermore, the corresponding thermal penetration depths are estimated to range between 25 and 50 µm^40^, which indicates that practically the entire donor ink thickness is heated up by the laser beam.

The hypothesis of pool-boiling is also supported by the relationship between these irradiation times (*t*_*i*_) and estimates of the characteristic times for boiling inception in the donor film. Assuming that the film is uniformly heated and ignoring thermal losses, the required time to trigger boiling (*t*_*B*_) can be estimated as:

2 $${t}_{B}=\frac{\pi {\rho }_{m}{D}^{2}l{c}_{p}({T}_{B}-{T}_{o})}{4P}$$

where *ρ*_*m*_ corresponds to the ink density, *l* to the donor film thickness, *c*_*p*_ to the specific heat of the ink, *T*_*B*_ to the boiling temperature, *T*_0_ to room temperature, and *P* to the laser power. For the ink and irradiation conditions of this work, *t*_*B*_ ranges between 2 and 8 μs^40^. Furthermore, the time required for a bubble to nucleate in these conditions (*t*_*N*_) can be estimated to be around 1 μs^40^. Both of these times (*t*_*B*_ and *t*_*N*_) are much shorter than the irradiation times *t*_*i*_, so that there is enough time to reach the boiling temperature and allow a large number of bubbles to nucleate during the scanning of the donor film.

The burst of the bubbles generated during pool-boiling would result in the ejection of material, which would in turn account for the spraying behavior. When the bubbles reach the liquid free surface, ink can be ejected through two successive mechanisms^48,49^. In the first place, the bubble burst proceeds through the breakup of the bubble dome wall into multiple fragments, which leads to the release of extremely small droplets. Next, the void left in the liquid free surface by each bursted bubble can also lead to a second ejection of droplets. These droplets would arise from the tiny jet that results from the streams converging in the bottom of the void when this is replenished. The perturbation of the liquid free surface originated by multiple consecutive bursts would result in the ejection of droplets in multiple directions, which would ultimately account for the observed spray.

From the images in Fig. 4 we can roughly estimate the liquid ejection speed in the following way: assuming a constant speed for the ejected droplets (at least during the early stages of emission), if we draw a right triangle whose hypotenuse is tangent to the spray front (Fig. 4, sketch displayed at 2.6 W, 450 mm/s), the speed can be inferred from the *x* and *z* measurements through the laser scan speed. The values obtained by this method are presented in Table 1 (in some cases the front is not clear enough to carry out a proper measurement). It can be observed that the ejection speed ranges between 0.1 and 1.2 m/s, with a trend to increase with scan speed for each laser power, and with no clear trend versus laser power for each scan speed.

**Table 1:** Estimated values of liquid ejection speed assuming it to be constant and using the triangle method.

Ejection speed (m/s)		Power (W)			
		1.0	2.0	2.6	3.6
Speed (mm/s)	150	0.1			
	300	0.3	0.3	0.4	
	450	0.8	0.5	0.7	0.8
	600	0.5	1.0	1.2	1.2

The front position was not enough well defined in some cases to provide a reasonably good estimate. In general, the ejection speed increases with laser scan speed, but no clear trend with laser power is observed.

**Figure 5:**
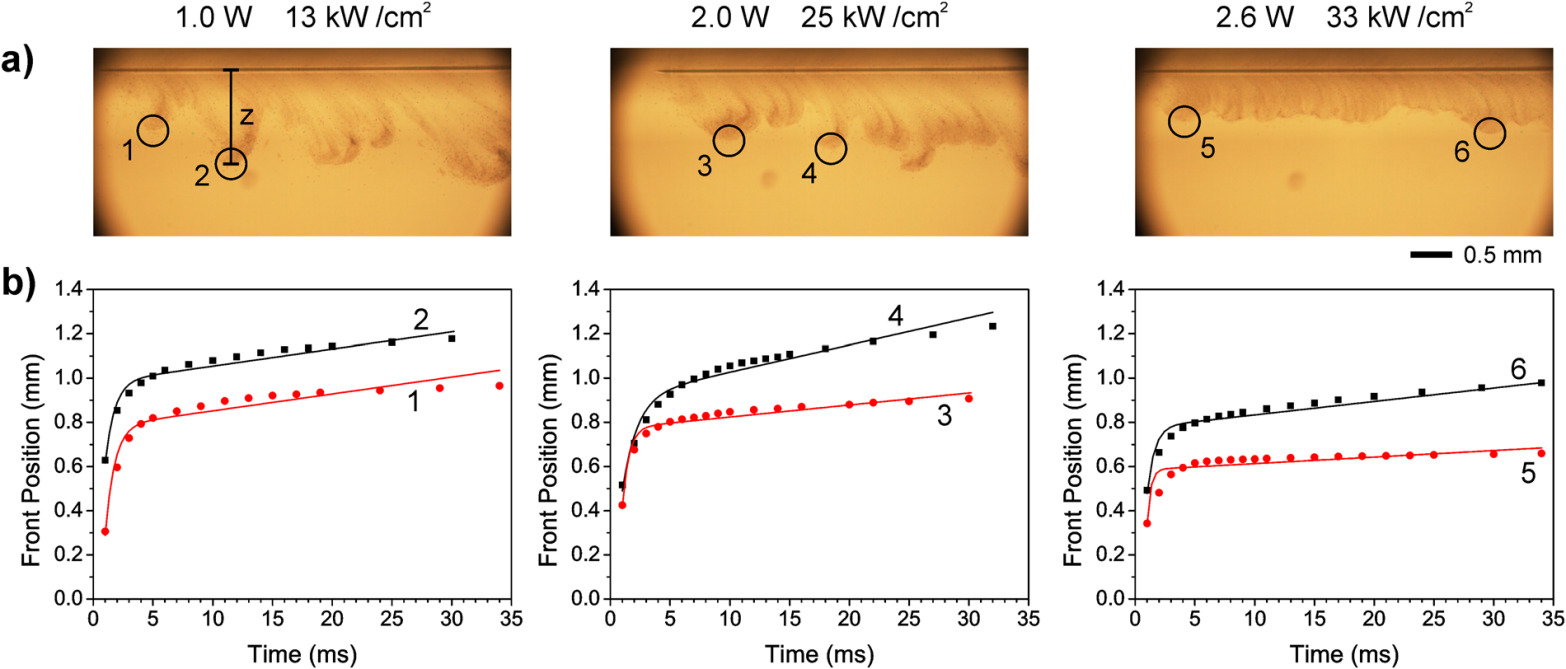
(**a**) Frames of the transfer dynamics corresponding to a scan speed of 300 mm/s and different laser powers (top). A circle indicates each of the lobes used for the plots in (**b**). (**b**) Plots of the front position of the selected lobes versus time (● and ■). All front position values have an associated uncertainty of 0.02 mm. It can be observed how the ink is rapidly ejected to further slow down to a constant terminal speed. Solid lines correspond to fits according to equation 4. The fit parameters are presented in Table 2.

The series of movies at a laser scan speed of 300 mm/s allows a more detailed analysis of the ejection speed than that performed in the former paragraph. It can be observed that liquid emission proceeds through a series of bursts that appear in the images as rather well-defined lobes, especially visible at low powers (1.0 and 2.0 W); lobes can also be appreciated at the other scan speeds for a laser power of 1.0 W, though they are not so apparent. As the power increases, the lobes evolve into a continuous and shorter curtain with a more uniform front. The evolution of the lobes can be easily tracked from the series of snapshots that constitute the movie (Supporting Information) since their shape does not change substantially as they propagate. Therefore, their vertical front position *z* with respect to the donor film surface can be easily measured at different times *t*, and from these measurements the *z(t)* dependence can be obtained. A couple of lobes were selected for each laser power (Fig. 5a) and their vertical front position was plotted versus time in Fig. 5b; in the case of 3.6 W it was not possible to accurately identify and track different lobes, so there is no plot for that power. In all the cases it can be observed how the droplets in the spray were initially ejected at a speed on the order of 1 m/s to very quickly slow down to a terminal velocity on the order of a few mm/s. The initial speeds obtained with this second method are between 3 and 4 times higher than the estimates presented in Table 1, measured with the less accurate method described in the preceding paragraph (which allows obtaining an average value for the early stages of ejection at best). Unfortunately, for the other laser scan speeds it is not possible to track the evolution of a well-defined expansion front allowing a measurement as accurate as that inferred from the plots in Fig. 5b. Nevertheless, the estimates of Table 1 at least provide an acceptable order of magnitude for the ejection speed at early times.

We can model the dynamic behavior of an ejected droplet in the spray front as that of a rigid sphere moving in air (and therefore submitted to both the force of gravity and a viscous frictional force) and not interacting with the other droplets around it. Assuming an average speed for the flying droplet of around 0.3 m/s (Table 1) and a diameter of around 5–10 µm, the corresponding Reynolds number associated with the air flow (density of 1.18 kg/m^3^ and dynamic viscosity of 1.85 × 10^−5^ kg/m·s at 300 K) ranges between 0.1 and 0.2. The values used for the diameter of the droplets in the calculation of the Reynolds number were chosen to be consistent with the sizes of the satellites in the border of the lines (around 20 µm); the diameter of a sessile droplet is between 2 and 4 times that of the spherical droplet with the same volume for a hydrophilic ink like the one used in the experiments. At the obtained Reynolds numbers, the Stokes law reasonably applies, and thus the viscous frictional force can be assumed to be proportional to the droplet speed according to the relation:

3 $${F}_{v}=3\pi \eta dv=kv$$

where *η* is the dynamic viscosity of air, *d* is the diameter of the droplet, and *v* its instantaneous speed. By solving the equation of motion in the *z* direction, the vertical position of the droplets in the front would be given by:

4 $$z(t)=\tau ({v}_{z0}-{v}_{z\infty })(1-{e}^{-(t-{t}_{0})/\tau })+{v}_{z\infty }(t-{t}_{0})$$

where *v*_*z0*_ is the *z* component of the ejection speed, *v*_*z∞*_ is the *z* component of the terminal velocity and *τ* is the damping time, defined as *τ* = *m/k* (where *m* is the droplets mass), and related to *v*_*z∞*_ by *v*_*z∞*_ = *gτ*, where *g* is the acceleration of gravity. For fitting purposes, an initial time *t*_0_ was considered in the equation, since the exact ejection time could not be determined with enough precision from the images. The parameters obtained from the fits are given in Table 2. As observed in Fig. 5b, the function given by equation 4 fits the experimental data points fairly well. The main discrepancy is found for a laser power of 2.6 W at early times, which results in an overestimation of the initial speed. This is clear in the comparison between images and plots in Fig. 5; for example, the values of *v*_*0z*_ and *v*_*z∞*_ for lobes 2 and 6 are similar, but the same *z* is attained for very different times. The slight departure of the fitted lines from the experimental plots can be attributed to the fact that 1) a flying droplet is not perfectly spherical and that 2) in the very early times of ejection the droplet speed is high enough for the Reynolds number to become close to 1, the limit of validity of Stokes law. In any case, the proposed model seems to describe quite well the observed spraying dynamics. Assuming that indeed the droplet motion is governed by Stokes’ law, the differences observed between lobes could be attributed to differences in droplet size, which would affect the *k* and *m* values. In fact, from the previous calculation we can determine the average diameter of the ejected droplets. Indeed, we can relate it with the terminal velocity *v*_*z∞*_ as:

5 $$d=3\sqrt{\frac{2\,\eta {v}_{z\infty }}{\rho g}}$$

where *ρ* is the density of the ink (1.3 kg/m^3^). The obtained values (Table 2) are in good agreement with the observed diameters of the sessile droplets in the borders of the lines, which provides additional support for the proposed model as a good description of the ejection dynamics corresponding to the sprayed droplets during CW-LIFT.

**Table 2:** Ejection speed (*v*_*z0*_), terminal velocity (*v*_*z∞*_) and damping time (*τ*) obtained from the fits of the plots in Fig. 5 according to equation 4.

Lobe number	Laser power (W)	*v*_*z0*_ (m/s)	*v*_*z∞*_ (mm/s)	*τ* (ms)	*d* (μm)
1	1.0	1.0	7.6	0.8	13
2		1.3	7.7	0.8	13
3	2.0	1.4	5.4	0.6	11
4		0.7	12.2	1.2	17
5	2.6	1.9	3.0	0.3	8
6		1.3	6.1	0.6	12

The droplet diameter (*d*) of the ejected droplets was estimated using equation 5.

The origin of the two different ejection modes, curtain of atomized ink and horsetail, seems difficult to determine. The experiment addressing this aim should encompass a close-up inspection of the donor film during irradiation, ideally one that allowed visualizing the onset of boiling in the ink bulk (hardly feasible in an opaque liquid like the used silver ink). Nevertheless, and even if only as a tentative hypothesis, the two ejection modes might be correlated with two different regimes characteristic of pool-boiling. The continuous spray corresponding to the horsetail mode seems compatible with the nucleate boiling regime, in which boiling proceeds through the formation of a large number of individual bubbles continuously bursting in the liquid surface. On the other hand, the presence of lobes in the curtain mode suggests that this mode could be associated with the transition boiling regime; in this regime individual bubbles coalesce into larger blobs whose intermittent burst could result in the observed lobes. However, aside from the mere qualitative correspondence outlined here, it seems difficult to correlate the proposed boiling regimes with the observed evolution of the ejection behavior with both laser power and scan speed. In addition to boiling, other effects (like the thermo-capillary flow described in the next section) contribute to the outcome of each ejection event, thus hindering the aimed correlation.

### Correlation between printed lines and transfer dynamics

The spraying dynamics seems at first quite incompatible with the printing of continuous lines like the ones obtained in the experiments. In the spray, the ejected droplets are scattered in all directions, so that it would be expected to find a wide and diffuse cloud of droplets on the receiver substrate instead of a rather well-defined line. However, the gap between donor and receiver is small enough (Fig. 4, sketch displayed at 3.6 W, 150 mm/s) to allow collecting most of the ejected material before there is substantial spreading. Nevertheless, some residual spread cannot be avoided, as is evident from the presence of the tiny satellite droplets in the line borders. Also due to the small gap, it can be understood that completely different ejection modes (curtain, horsetail) can lead to similar printed lines; see, for example, the lines and associated ejection dynamics corresponding to 1.0 W, 300 mm/s (curtain) and 2.0 W, 450 mm/s (horsetail) in Fig. 4. Thus, there seems to be no correlation between these two types of ejection modes and the two line morphologies obtained when there is significant amount of transferred material (uniform lines and lines with a darkened center); naturally, the almost invisible traces correspond to the absence of visible ejection in the fast-photography movies.

The evolution of both the printed line morphology and ejection dynamics with both laser power and scan speed is puzzling. We observe that the amount of transferred ink decreases when the laser power increases and the scan speed decreases (Fig. 2a). In both situations the energy per unit area accumulated during the scan increases, so that one would expect an increase in the amount of deposited material, contrarily to what really occurs. Furthermore, in this evolution ink depletion starts from the center of the lines towards the edges (lines with darkened center), until no material is found on the receiver substrate (3.6 W, 150 mm/s). A similar trend is observed in the fast-photography images (Fig. 4): fewer material seems to be ejected as the accumulated energy per unit area increases, though for 450 and 600 mm/s the evolution is not so clear.

The evolution with both laser power and scan speed of the printed line morphology could be easily explained assuming that the deposited ink is vaporized again by the scanning laser beam. This would be consistent with the long confocal parameter (2.2 mm) of our laser system, and with the Gaussian distribution of our laser beam, that would account for the darkened center of the lines. However, this hypothesis is not compatible with the evolution observed during material ejection: at high laser powers and low scan speeds there is practically no material transfer. Therefore, another explanation is required.

**Figure 6:**
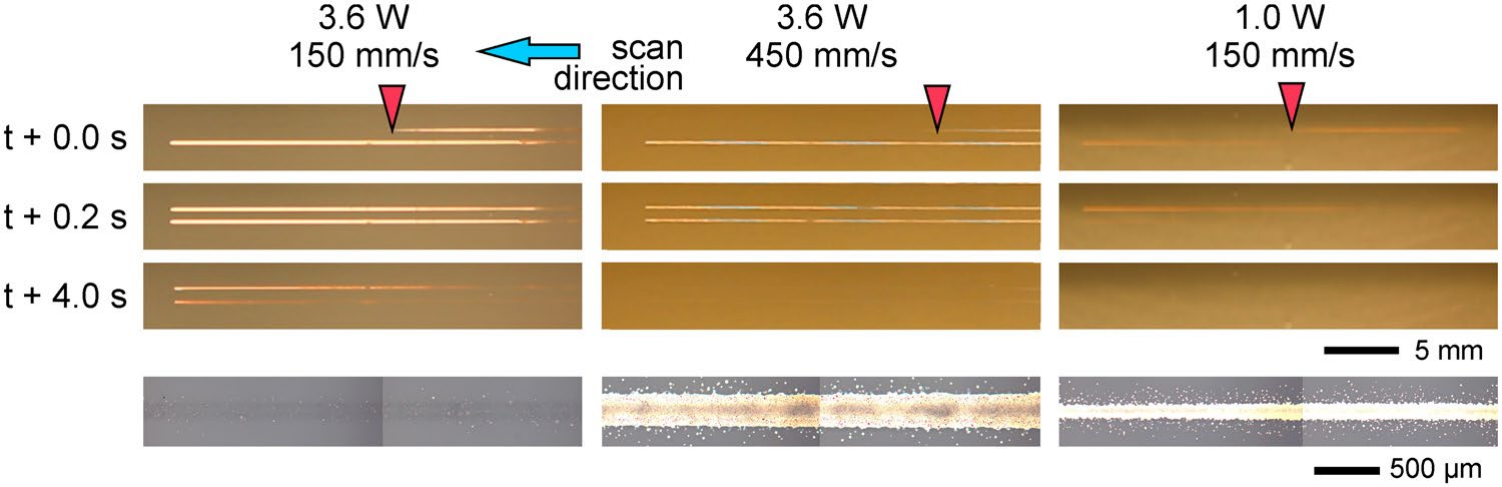
High-angle view of the donor film surface as the laser beam scans it at different laser powers and scan speeds; two parallel lines recorded at different times are presented in each frame. The line depletion and recovery time in the donor film is more prominent at slow scan speed and high laser power. Optical microscopy images of the corresponding printed lines are also presented.

In order to provide the aimed explanation, we recorded a high-angle movie of the evolution of the donor film surface as the laser beam was scanned along it. A selection of frames obtained at conditions leading to the three types of line morphology is presented in Fig. 6. The selection displays the transfer of two parallel lines at different times during the scanning process. It is observed that the laser scan depletes ink in the lines, and that ink depletion is more prominent as the accumulated energy per unit area increases; furthermore, it takes longer for the line to replenish. The depletion, however, cannot be attributed completely to the ejection of ink, since in the conditions where it is most prominent (3.6 W, 150 mm/s), no material ejection is observed in the fast-photography images (Fig. 4). It can be attributed, however, to thermo-capillary convection, a phenomenon commonly observed in the sintering of inks using CW laser irradiation^44,50,51^. As the Gaussian laser beam scans the donor film it locally heats up the ink, creating a temperature gradient between the irradiated region and the rest of the film. This gradient results in an outwards flow of ink away from the beam spot. The thermo-capillary convection hypothesis can also explain the evolution observed from one type of line morphology to another. At 3.6 W and 150 mm/s thermo-capillary convection is maximum, which results in ink depletion in the totality of the irradiated area, so that no ink is ejected and no material is deposited accordingly (Fig. 4). When the scan speed is increased to 450 mm/s at the same laser power (lower accumulated energy per unit area), ink depletion is mitigated and occurs mainly in the center of the line (peak of the Gaussian distribution). This can account for the line with darkened center obtained in these conditions: ink is mostly ejected from the borders of the line. This effect could even be enhanced by similar convection in the deposited ink induced by the laser beam, whose long confocal parameter would allow it to reach the receiver substrate^44^. Finally, at the mildest conditions (1.0 W, 150 mm/s) we observe very little depletion (Fig. 6), if any, which indicates a weak contribution of thermo-capillary convection. In the practical absence of ink depletion, all the irradiated area contributes to pool-boiling, which results in the obtained uniform line.

A simple calculation can help to test the consistency of the thermo-capillary flow hypothesis. The driving force in any Marangoni flow is the surface tension gradient that, assuming it to be constant, can be approximated by $\frac{{\rm{\Delta }}\sigma }{D/2}$, where *Δσ* corresponds to the surface tension difference between the center of the laser scanned line and its border (*D* is the diameter of the laser beam). This surface tension gradient must balance the tangential stress *τ*, which in a flow parallel to the liquid free surface with a velocity profile *u(z)* (with *z* the Cartesian coordinate perpendicular to the liquid free surface) is given by:

6 $$\tau =\mu \frac{\partial u}{\partial z} \sim \mu \frac{U}{l}$$

where *μ* is the dynamic viscosity of the liquid and *U* the flow velocity in the liquid surface (*l* is the thickness of the donor film). The approximation in equation 6 assumes that the ink is at rest at the donor film-substrate interface. The combination of equation 6 and the surface tension gradient estimate allows obtaining the capillary flow velocity:

7 $$U \sim \frac{{\rm{\Delta }}\sigma }{\mu }\frac{2l}{D}$$

In thermo-capillary convection the surface tension gradient is generated by a temperature gradient, in our case the temperature difference between the center of the laser scanned line and its edge. For our water-based ink, we can assume *Δσ* to be around 13 mN/m^52^, assuming that the center of the line is at the boiling temperature of water and that the edge remains at room temperature, 300 K. Using the parameters provided in the Methods section, *U* results to be on the order of 1 m/s. We can then obtain a characteristic time (${t}_{c}=\frac{D/2}{U}$) for this process of around 50 μs, smaller than the irradiation times *t*_*i*_ found in the previous section, which makes it plausible that thermo-capillary flow is responsible for the ink depletion observed in the laser scanned lines (Fig. 6).

It might be tempting to compare the characteristic time *t*_*c*_ with the boiling trigger time *t*_*B*_ obtained in the preceding section in order to better test the thermo-capillary flow hypothesis; in fact, the presence or absence (total or partial) of material in the printed lines will depend on the competition between boiling and capillary convection. However, such comparison would not be completely correct. First, it is important to stress that both times, *t*_*c*_ and *t*_*B*_, are only rough estimates, too rough to withstand an accurate comparative analysis. Second, *t*_*B*_ is the time required to start bubble nucleation in the donor film, but the onset of massive boiling in the liquid bulk (needed for droplets ejection) does not occur until a substantially longer delay, hard to estimate. Third, *t*_*c*_ is the time required for the convection front to reach the border of the line, but partial ink depletion can start earlier than that. In fact, according to the result of equation 7, a stripe around 20 µm thick in the center of the line could be depleted of ink due to thermo-capillary flow in only 10 µs. A final remark is still in order. The estimate of equation 7 is independent of both the laser power and the laser scan speed, but the experimental results are clearly not. This is a consequence of using a linear surface tension profile in the calculations: any other profile (Gaussian, for example) has non-constant gradient, and would therefore be dependent on the irradiation conditions.

## Conclusions

The study of the influence of laser power and scan speed on the morphology of the CW-LIFT printed outcomes has revealed a range of conditions where the technique is feasible for printing continuous lines of conductive inks free from bulging. The study has also brought to light the surprising evolution of the amount of transferred material versus printing conditions: above a certain threshold, it decreases with increasing laser power and decreasing scan speed. The optimum printing conditions correspond to relatively low laser powers and high scan speeds, the same conditions at which the lowest sheet resistances are obtained.

The fast-photography investigation of the liquid ejection process has shown that transfer proceeds through a spraying behavior that clearly contrasts with the jetting dynamics typical of pulsed LIFT. The images have revealed two different modes of spray depending on the irradiation conditions: a curtain of atomized ink at low scan speeds, and a horsetail-shaped spray predominantly at high scan speeds. The estimated time scales involved in the process are consistent with the hypothesis of pool-boiling in the donor film as the mechanism responsible for the observed spray. The motion of the sprayed droplets can be well described with the simple model of a rigid sphere moving in air submitted to the action of gravity and viscous drag. The assessment of the droplet terminal velocity allows estimating their size, which is in good agreement with that observed in the deposits.

In spite of the broad angle of ejection characteristic of the spraying dynamics, it is possible to attain rather good definition in the printed lines by setting a small gap between donor and receiver substrates. Nevertheless, some tiny satellites are always present in the edges of the lines. The combined analysis of the transfer dynamics with the morphology of the printed lines has provided a plausible explanation for the surprising evolution of their thickness with laser power and scan speed: thermo-capillary flow in the donor film results in increased ink depletion in the scanned line at irradiation conditions of high accumulated energy per unit area.

## Methods

### Laser direct-writing system

All the experiments were carried out using an Nd:YAG laser (Baasel Lasertech, LBI-6000) working at the fundamental wavelength (1064 nm) in CW mode. The beam had a Gaussian intensity profile with a maximum output power of 5 W. The laser was equipped with a set of two galvanometric mirrors which allowed to scan the laser beam along the sample at speeds ranging from 1 to 600 mm/s. After the galvo head an f-theta lens (100 mm) focused the laser beam on the sample plane. The resulting beam diameter on the donor film was around 100 μm.

### Sample preparation and printing

The transferred ink was a commercial silver nanoparticle water-based ink (Metalon® JS-B25HV) commonly used in inkjet printing applications, with a viscosity of 8 mPa·s, a density of 1.3 g/cm^3^ and a solid content around 25% in weight (particle size smaller than 60 nm). The ink was spread along the donor substrate, a glass microscope slide, using a blade coater, ensuring a thickness of about 30 μm (estimated through weight measurements); the receiver substrate was also a conventional microscope glass slide. A gap of 150 μm between donor and receiver substrates was set through spacers in order to ensure proper transfer. The sample was placed on a hot plate that was kept at 75 °C; it helped to pin the contact line of the liquid once the ink was transferred. After transfer, the Ag-NP lines were left to dry on the hot plate and subsequently cured in an oven for 1 hour at 200 °C.

### Sample characterization

Line characterization was carried out using optical microscopy (Carl Zeiss, model AX10 Imager.A1), confocal microscopy (Sensofar PLμ 2300) and scanning electron microscopy (JEOL J-7100). Electrical measurements of sheet resistance were carried out using a two point probe.

### Fast-imaging system

The imaging setup consisted on a fast-camera (AOS Technologies AG, model S-PRI F1), with an acquisition speed of 1000 frames per second, coupled to a 10× microscope objective (numerical aperture of 0.28), and a 150 W halogen light source (ThorLabs Inc, model OSL1-EC) facing the camera in shadowgraphy configuration^22,23,53^. The sample was placed between the objective and the lamp at grazing incidence respect to the optical axis, so that the donor substrate appeared on top of the recorded image with the ink film facing downwards. The receiver substrate was removed during image acquisition.

## Electronic supplementary material

Ejection dynamics at 1.0 W and different scan speeds

Ejection dynamics at 2.0 W and different scan speeds

Ejection dynamics at 2.6 W and different scan speeds

Ejection dynamics at 3.6 W and different scan speeds
